# Decoding neurovascular signatures: advanced imaging insights in DADA2-Related Cerebral Microangiopathy

**DOI:** 10.1007/s10072-025-08676-9

**Published:** 2025-12-19

**Authors:** Yaping Zhou, Min Shen, Nan Jiang, Hanhui Fu, Fei Han, Yi-Cheng Zhu, Ming Yao, Jun Ni

**Affiliations:** 1 Department of Neurology, State Key Laboratory of Complex Severe and Rare Diseases, Dongcheng District, Peking Union Medical College Hospital, Chinese Academy of Medical Sciences and Peking Union Medical College, No.1 Shuaifuyuan, DongdanBeijing, 100730 China; 2 Department of Rare DiseasesState Key Laboratory of Complex Severe and Rare DiseasesDepartment of Rheumatology and Clinical ImmunologyNational Clinical Research Center for Dermatologic and Immunologic Diseases (NCRC-DID), Ministry of Science & TechnologyKey Laboratory of Rheumatology and Clinical Immunology, Ministry of Education, Peking Union Medical College Hospital (PUMCH), Chinese Academy of Medical Sciences & Peking Union Medical CollegePUMCHPUMCH, Beijing, 100730 China

**Keywords:** Deficiency of adenosine deaminase 2, Neuroimaging tirad, Microangiopathy, Inflammation, Mechanism

## Abstract

**Background and purpose:**

Neuroimaging phenotypes remain poorly characterized in patients with deficiency of adenosine deaminase 2 (DADA2). We aimed to characterize cerebral microangiopathy patterns and establish diagnostic imaging biomarkers for DADA2.

**Methods:**

We retrospectively analyzed consecutively enrolled DADA2 patients with neurological involvement from two ongoing prospective cohorts. The demographic, clinical, and neuroimaging data were evaluated, focusing on neuroimaging lesion patterns and cerebral small vessel disease (CSVD) imaging markers.

**Results:**

Thirteen patients were included, with 69.2% males. The median age at neurological onset was 17 (7–26) years. A striking disparity emerged between acute ischemic burden and chronic small vessel injury markers. During a follow-up of 51 (0.5–193) months,138 ischemic lesions were observed in all patients, primarily involving the pons (38 lesions), thalamus (21 lesions), internal capsule (20 lesions), and basal ganglia (18 lesions). Conversely, only one cerebral microbleed (CMB) was detected in all patients. Mild white matter hyperintensities (WMH) were observed in 9 patients. Infratentorial atrophy was prevailing and present in 69.2% (n = 9) patients. Additionally, hemorrhagic stroke was less frequent (n = 6, 46.2%) than infarction.

**Conclusion:**

DADA2 exhibited a profoundly high risk of stroke recurrence, with substantial lesions being clinically asymptomatic, underscoring the importance of routine neuroimaging evaluation for DADA2 patients. This disease presents a unique CSVD phenotype characterized by brainstem-deep gray nucleus ischemic predominance and infratentorial atrophy yet paradoxically low burdens of CMBs and WMH compared to other CSVD. This distinctive imaging triad not only facilitates early recognition and timely intervention, but also suggests a pathophysiological divergence from other CSVD.

**Supplementary Information:**

The online version contains supplementary material available at 10.1007/s10072-025-08676-9.

## Introduction

Deficiency of adenosine deaminase 2 (DADA2), first described in 2014, is a rare monogenic autoinflammatory disorder caused by loss-of-function mutations in the *ADA2* gene (also known as *CECR1*) [1, 2]. This multisystem pleiotropic disease classically manifests as a triad of systemic vasculitis, hematological abnormalities, and immunodeficiency [3, 4], with emerging evidence implicating dysregulated interferon signaling and neutrophil extracellular trap formation in its pathogenesis. Most patients experience disease onset before the age of 10 years, though adult-onset cases are increasingly recognized, broadening the diagnostic spectrum [5]. Characterized by multiorgan involvement and significant mortality (exceeding 8% by age 30) [6], DADA2 poses diagnostic challenges due to its striking phenotypic heterogeneity [4]. Moreover, neurological involvement is prevalent in DADA2 with stroke recognized as the leading cause of mortality [1, 2, 7, 8]. Given the marked efficacy of anti-tumor necrosis factor (TNF) agents in halting disease progression [9–11], early recognition and diagnosis are crucial for optimizing outcomes. This diagnostic complexity and the imperative for early diagnosis and timely intervention underscore the urgent need for comprehensive phenotypic characterization, particularly regarding neurological manifestation.

Neurological manifestations affect 50–77% of DADA2 patients, with early-onset lacunar stroke (typically occurring before age 30) representing a hallmark feature [8, 12–15]. Notably, the stroke recurrence rate in DADA2 approaches 76%, substantially exceeding that of conventional young-onset stroke etiologies [10]. Pathologically, DADA2-associated vasculopathy targets small- to medium-sized arteries, characterized by interstitial neutrophil and macrophage infiltration and non-granulomatous necrotizing arteritis [16, 17]. Consequently, DADA2 should be considered in young patients with recurrent strokes and systemic inflammation. Notably, transient or mild neurological symptoms may precede overt infarcts, potentially delaying diagnosis. Recognition of neuroimaging features and disease-specific imaging markers is therefore essential for early identification and timely treatment.

However, investigations of the neuroimaging features in DADA2 are limited [18, 19]. Moreover, although DADA2 is considered a monogenic cerebral small vessel disease (CSVD), the characteristics of CSVD imaging markers in this disorder are poorly defined [15]. Therefore, to address this issue, we analyzed the neuroimaging features, including the lesion types, spatial distributions, temporal evolutions, and characteristics of CSVD imaging markers in 13 DADA2 patients, aiming to improve the differentiation from other CSVD entities.

## Methods

### Study subjects and data collection

We retrospectively enrolled 13 patients with neurological involvement from two ongoing prospective cohorts at Peking Union Medical College Hospital from March 2015 to February 2025. The diagnosis of DADA2 was confirmed by both identification of pathogenic variants in the *ADA2* gene through sequencing and demonstration of reduced ADA2 enzymatic activity in all patients, except for one individual diagnosed solely based on genetic confirmation of compound heterozygous pathogenic *ADA2* mutations (Table S1 in the supplementary material).

The demographic and clinical information, including age, sex, symptoms, and signs, was collected from the hospital information system. Acute-phase reactants, specifically the erythrocyte sedimentation rate (ESR) and high-sensitivity C-reactive protein (hsCRP), were assessed in all patients during their hospitalization. Laboratory results were summarized for 12 patients who had not received TNF inhibitor therapy prior to admission. All available neuroimaging data of the 13 patients were collected for analysis. All the patients underwent abdominal ultrasound or CT scans. Specifically, five patients underwent abdominal CT scans, three patients underwent abdominal ultrasound, and five patients underwent both modalities.

Informed consent was obtained from all the participants or their legal surrogates. The study was approved by the Ethics Committee of the Peking Union Medical College Hospital (JS-1280 and ZS-3272).

### Neuroimaging evaluation

Brain magnetic resonance imaging (MRI) examinations were conducted for all 13 patients. The MRI scans were performed on 3.0 T scanners according to the standardized imaging protocols with the following sequences: 1) axial and sagittal T1-weighted imaging (T1WI); 2) axial and sagittal T2-weighted imaging (T2WI); 3) axial fluid-attenuated inversion recovery (FLAIR) imaging; and 4) axial diffusion-weighted imaging (DWI) and apparent diffusion coefficient (ADC) imaging; 5) susceptibility-sensitivity weighted images (SWI) or T2*-weighted imaging. SWI, which is more sensitive for microbleed detection, was performed in 12 patients, while 1 patient was evaluated with T2*-weighted imaging only. To further validate the neuroimaging features identified via 3.0 T MRI, high-field 5.0 T MRI examinations were additionally performed in 6 patients, enabling enhanced spatial resolution for precise characterization of microstructural abnormalities. Moreover, 3 patients underwent spinal MRI examinations with axial and sagittal T1WI, T2WI, and T2WI with fat saturation sequences. Cerebral angiographies, including magnetic resonance angiography (MRA), computed tomography angiography (CTA), digital subtraction angiography (DSA), and high-resolution MRI (HRMRI) of the vessel walls, were also conducted for 10 patients. The specific neurovascular imaging modalities employed were as follows: five patients underwent MRA alone, two underwent CTA alone, and one underwent HRMRI alone. Additionally, one patient received both CTA and DSA, and another received both CTA and HRMRI.

The neuroimaging results were independently evaluated by two experienced neurologists (MY and YPZ, with 20 and 5 years of experience, respectively). Ischemic and hemorrhagic lesions were documented for their presence and anatomic distribution. Imaging biomarkers of CSVD were systematically analyzed, including cerebral atrophy, cerebral microbleeds (CMBs), periventricular white matter hyperintensities (pWMH), and deep white matter hyperintensities (dWMH). The CMBs and WMH were identified according to STRIVE-2 standards [20]. Specifically, WMH was characterized by hyperintensity on T2WI and FLAIR without cavitation, while CMBs were detected on SWI or T2* and characterized by small round or ovoid hypointensity with blooming. The WMH burden was semiquantitatively graded using the Fazekas scale. Longitudinal changes in lesions extent, quantity, and signal intensity of lesions were assessed among patients with serial MRI examinations. Angiographic data(e.g., aneurysms, stenosis, and other abnormalities) and spinal cord abnormalities (when spinal MRI was available) were also assessed.

### Statistical analysis

Continuous data were expressed as the median (minimum to maximum), and the categorical data were described using frequencies and percentages.

## Results

### Demographic and clinical characteristics

Demographic and clinical characteristics are summarized in Table 1. Among the 13 patients, a male predominance was observed (69.2%, 9/13) with females comprising 30.8% (4/13) of the cohort. The median age at disease onset was 16 (0.5–26) years, and the median diagnostic delay spanned 11 (0–18) years, resulting in a median age at confirmed diagnosis of 23 years (range 16–38). Notably, neurological manifestations emerged at a median age of 17 years (range 7–26), underscoring the early neurovascular involvement.

**Table 1:** Demographics and clinical features of the patients

	Overall (n = 13)
Age at onset (years, median, range)	16 (0.5–26)
Age at diagnose (years, median, range)	23 (16–38)
Time from onset to diagnose (years, median, range)	11 (0–18)
Age at neurological onset (years, median, range)	17 (7–26)
Time for follow up† (month, median, range)	51 (0.5–193)
Males, n (%)	9 (69.2)
Fever, n (%)	9 (69.2)
Skin involvement, n (%)	
Livedo reticularis	10 (76.9)
Skin/vulvar ulcers	3 (23.1)
Raynaud's phenomenon	3 (23.1)
Erythema modosum	3 (23.1)
Ophthalmologic involvement*, n (%)	8 (61.5)
Hepatosplenomegaly, n (%)	10 (76.9)
Hypertension, n (%)	4 (30.8)
Hematological involvement^&^, n (%)	3 (23.1)
Initial symptom, n (%)	
Skin involvement	6 (46.2)
Stroke	5 (38.5)
Fever	1 (7.7)
Hearing loss	1 (7.7)
Neurological involvement, n (%)	
Ischemic stroke	11 (84.6)
Hemorrhagic stroke^#^	6 (46.2)
Spastic paraplegia	2 (15.4)
Headache	2 (15.4)
Hearing loss	4 (30.8)
Total number of ischemic strokes	41
Number of ischemic strokes per patient (median, range)	3 (0–9)
Laboratory results^§^, n (%)	
Elevated ESR	5 (41.7)
Elevated hsCRP	8 (66.6)

^†^ A total of 12 patients were available

^*^ The ophthalmologic involvements included retinal vasculitis in 3 patients, dry eye disease in 2 patients, central retinal artery occlusion in 1 patient, oculomotor nerve palsy in 1 patient, and optic neuropathy in 1 patient

^#^ Hemorrhagic stroke included intracranial hemorrhagic stroke in 5 patients and spinal hemorrhage in 1 patient

^&^ Including 1 patient with thrombocytopenia, leukopenia, and anemia, 2 patients with anemia and mild thrombocytosis

^§^Data available for 12 patients collected before anti-TNF therapy

Abbreviations: ESR: erythrocyte sedimentation rate, hsCRP: high sensitivity C-reactive protein, TNF: tumor necrosis factor

Ischemic stroke predominated, affecting 11 (84.6%) patients with recurrent episodes in 9 (69.2%). A total of 41 symptomatic ischemic strokes were documented in our cohort, with a median of 3 attacks per patient (range 0–9). Hemorrhagic strokes were observed in 6 (46.2%) patients, involving cerebral regions in 5 cases (38.5%) and spinal cord in 1 case (7.7%). Moreover, all hemorrhagic events coexisted with ischemic strokes. Additional manifestations included spastic paraplegia (n = 2, 15.4%), hearing loss (n = 4, 30.8%), and headache (n = 2, 15.4%).

### Neuroimaging findings

The neuroimaging characteristics of the cohort are summarized in Table 2.

**Table 2:** Summary statistics of neuroimaging findings in DADA2 patients

Characteristics	All patients (N = 13)
Ischemic lesions	13 (100%)
Hemorrhagic lesions*	6 (46.2%)
Both ischemic and hemorrhagic lesions	6 (46.2%)
Ischemic lesion locations	
Brain stem	11 (84.6%)
Midbrain	5 (38.5%)
Pons	10 (76.9%)
Medulla	7 (53.8%)
Thalamus	10 (76.9%)
Basal ganglia	7 (53.8%)
Internal capsule	9 (69.2%)
Callosum	3 (23.1%)
Periventricular regions	6 (46.2%)
Subcortical white matter	3 (23.1%)
Hemorrhagic lesion locations	
Temporal lobe	2 (15.4%)
Frontal lobe	1 (7.7%)
Basal ganglia	3 (23.1%)
Spine	1 (7.7%)
CSVD imaging biomarkers	
CMBs	1 (7.7%)
PWMH	9 (69.2%)
DWMH	4 (30.8%)
Atrophy	9 (69.2%)
Asymmetric atrophy	4/9 (44.4%)
Symmetric atrophy	5/9 (55.6%)
Follow up	
Increased numbers or range of lesions	11/12 (91.7%)
Regression or vanished	3/12 (25.0%)
Unchanged	1/12 (8.3%)

^*^ Hemorrhagic lesions included intracranial hemorrhagic lesions in 5 patients and spinal hemorrhage in 1 patient

The denominator indicates the available case numbers

Abbreviations: CMBs: cerebral microbleeds, DWMH: deep white matter hyperintensities, PWMH: periventricular white matter hyperintensities

#### Predominance of Ischemic lesions over sparse CMBs and WMH

Ischemic lesions constituted the predominant neuroimaging finding observed in all patients, with a total of 138 lesions identified, predominantly manifesting as lacunes or acute lacunar infarcts. Lacunes were defined according to STRIVE-2 standards [20], presenting as hyperintensity on T2WI, hypointensity on T1WI, and hypointensity on FLAIR imaging with a hyperintense rim.

**Fig. 1:**
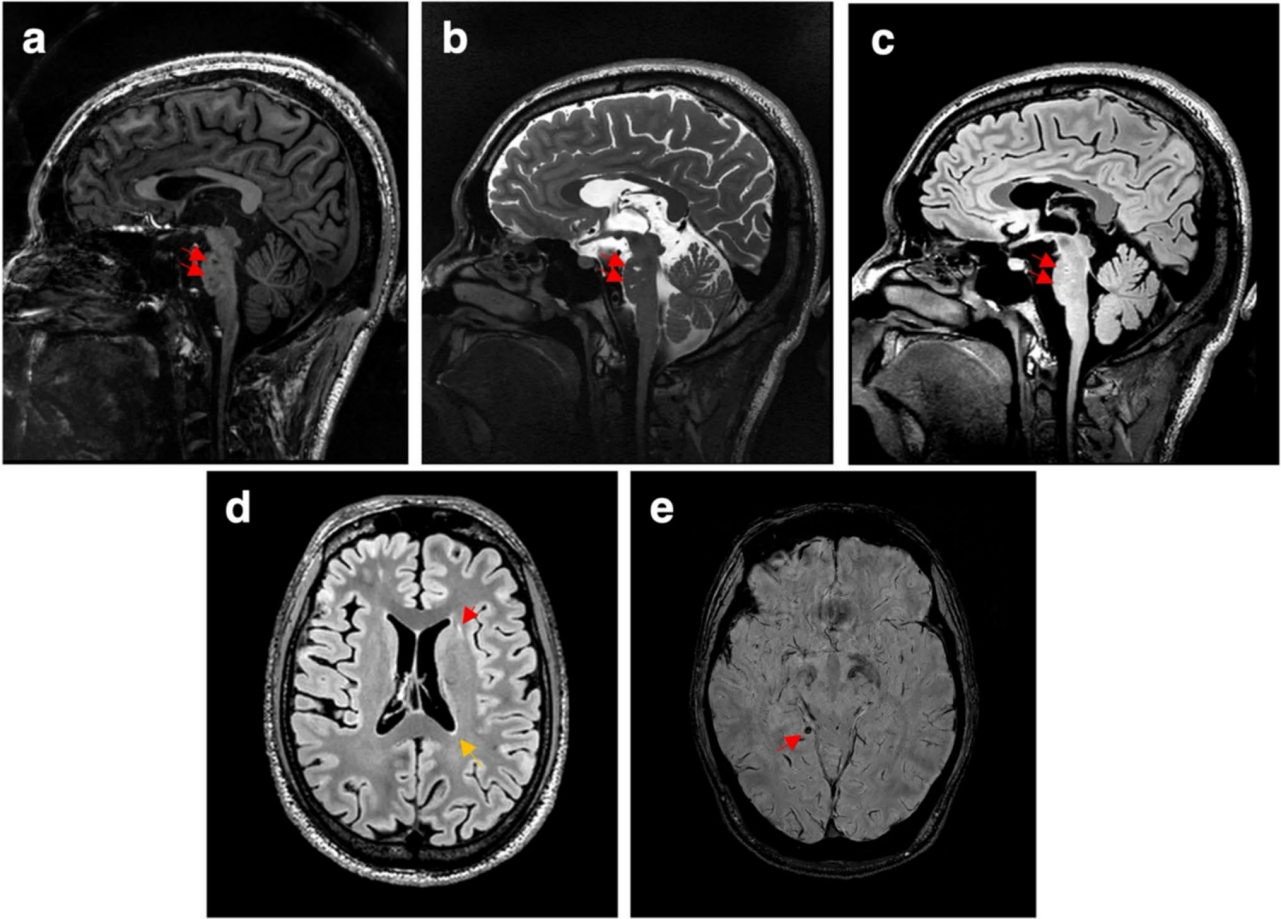
The CSVD imaging markers in DADA2 on 5.0 T MRI. a-c: The sagittal T1WI (**a**), T2WI (**b**), and FLAIR (**c**) show multiple lacunes (arrow) on the brainstem, and atrophy of the brainstem and cerebellum. d: The axial FLAIR shows dWMH (red arrow) and pWMH (yellow arrow). e: The SWI shows a CMB (arrow). All the imaging were from a 22-year-old male, 1 month after anti-TNF therapy. Abbreviations: CMB: cerebral microbleed, CSVD: cerebral small vessel disease, DADA2: Deficiency of adenosine deaminase 2, dWMH: deep white matter hyperintensities, FLAIR: fluid-attenuated inversion recovery imaging, MRI: magnetic resonance imaging, pWMH: periventricular white matter hyperintensities, T1WI: T1-weighted imaging, T2WI: T2-weighted imaging, SWI: sensitivity weighted images.

CMBs were exceptionally uncommon, with only a single CMB observed in one patient (7.7%) across the entire cohort. Minimal burdens of dWMH and pWMH were observed in our cohort, with all cases exhibiting Fazekas grade 1 except one patient with Fazekas grade 2 (Fig. 1). The prevalence of dWMH was 30.8% (n = 4), while pWMH was observed in 69.2% (n = 9) of the cases. Longitudinal neuroimaging assessments demonstrated no significant progression of CMBs or WMH burden over a median follow-up period of 51 months (range 0.5–193).

#### Distributions and characteristics of the ischemic lesions

**Fig. 2:**
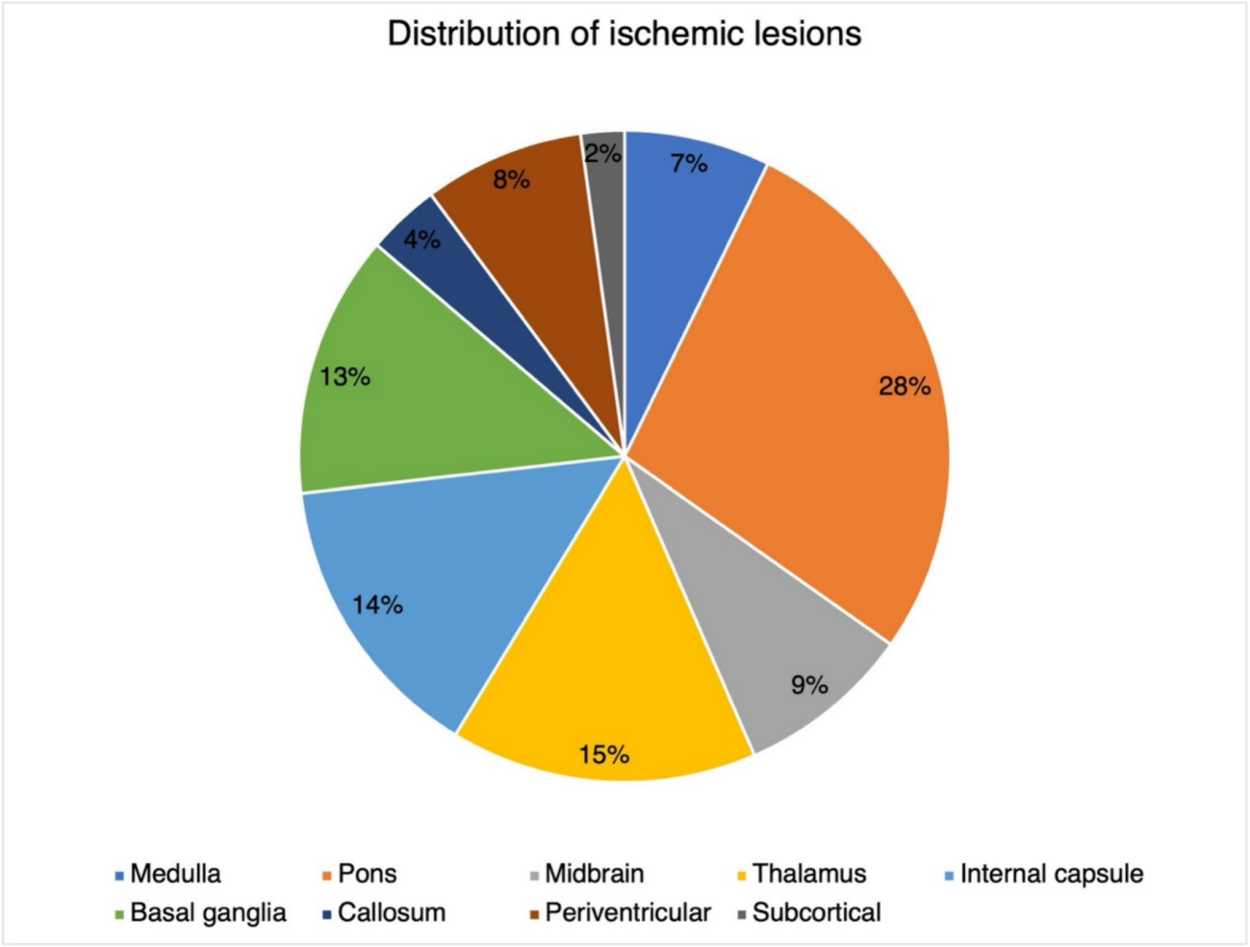
The distribution of ischemic lesions in DADA2. In the cohort of 13 patients, a total of 138 infarcts were observed. The lesions were primarily located in the brainstem and thalamus. Abbreviations**:** DADA2: Deficiency of adenosine deaminase 2.

The cohort exhibited a substantial ischemic burden, with 138 lesions identified across 13 patients. Lesion distribution followed distinct anatomical patterns: predominantly localized to the pons (38 lesions, 10 patients), followed by the thalamus (21 lesions, 10 patients), internal capsule (20 lesions, 9 patients), basal ganglia (18 lesions, 7 patients), and other regions (Fig. 2). Most patients (12/13, 92.3%) presented multiple ischemic lesions.

Of all the 138 ischemic lesions, 50 (36.2%) were acute lacunar infarcts or lacunes on 3.0 T MRI. The remaining 88 (63.8%) lesions appeared hyperintense on T2WI and hypointense on T1WI, with or without hyperintense in FLAIR imaging on 3.0 T MRI. Notably, all the lesions were definitively characterized as lacunes on 5.0 T MRI, which provided superior resolution for detecting small lesions compared to 3.0 T MRI (Fig. S1 in the supplementary material).

The total number of lesions exceeded the number of clinical events. Only 45 lesions (32.6%) correlated temporally with acute symptomatic strokes, while 93 (67.4%) were asymptomatic.

#### Ischemic Lesion Chronoarchitecture

Twelve patients were followed up for a median of 51 months (range 0.5–193). Longitudinal MRI analysis of these patients demonstrated progressive ischemic burden in 91.7% (11/12), while 8.3% (1/12) remained radiologically stable. All 12 patients received anti-TNF therapy following the diagnosis of DADA2, with all patients achieving neurological symptom stabilization after treatment. Of the 12 patients, post-treatment neuroimaging evaluations were available for 6 patients, revealing complete lesion stabilization. Acute infarcts evolved into chronic lacunar cavities in all cases, though partial regression was observed in 3 patients. Moreover, one lacunar infarct lesion resolved completely on both 3.0 T and 5.0 T MRI in 1 patient (Fig. S2 in the supplementary material).

#### Infratentorial rather than supratentorial volume Loss

Infratentorial atrophy involving the brain stem, cerebellum, and cerebral peduncle, was prevalent in 69.2% (9/13) patients, invariably coexisting with strategic ischemic/hemorrhagic lesions in the brainstem or basal ganglia, independent of WMH burden. Symmetrical atrophy was detected in 5 of the 9 patients (55.6%) and was exacerbated by the accumulation of ischemic lesions. Asymmetric atrophy was observed in 44.4% (n = 4 of 9) patients, potentially attributable to Wallerian degeneration and aggravated after cerebrovascular events (Fig. S3 in the supplementary material).

#### Hemorrhagic lesions and angiographic results.

Compared with ischemic lesions, symptomatic hemorrhagic lesions (including hematoma and apoplectic cyst, excluding CMBs) were relatively uncommon, affecting 46.2% (6/13) of the cohort. The hemorrhagic lesions were distributed in the cerebral lobes (3 lesions, 3 patients), deep (3 lesions, 2 patients), and spinal cord (1 lesion, 1 patient). Patients with hemorrhagic lesions demonstrated higher frequencies of fever (83.3% vs. 57.1%) and hematological involvement (50.0% vs. 0%) compared to those without hemorrhagic lesions (Table S2 in the supplementary material). Of the 6 patients who presented hemorrhagic lesions, 1 patient was taking warfarin, while 2 patients were taking aspirin prior to the hemorrhage. The agents were prescribed for secondary prevention following recurrent ischemic stroke. The remaining 3 patients had no documented usage of antiplatelet or anticoagulation agents.

Cerebral angiographic data were available in 10 patients. Multiple intracranial microaneurysms were detected in 1 patient (10.0%), cavernous hemangioma was observed in 1 patient (10%), and segmental stenosis of intracranial arteries was found in 1 patient (10.0%). HR-MRI of the vessel wall was available for 2 patients with normal presentations. The angiographic abnormalities were unrelated to their clinical events.

## Discussion

The present study, representing the largest and the most comprehensive neuroimaging investigation of DADA2 patients, systematically delineated the CSVD imaging markers and temporal evolution of lesions. Our findings reveal a distinct neuroimaging triad: 1) brainstem-deep gray matter ischemic predominance, 2) infratentorial atrophy, and 3) paradoxically low burdens of CMBs and WMH compared to other CSVDs. This signature may suggest a different pathophysiological mechanism of DADA2 from other CSVD, and further redefine DADA2 as a distinct cerebrovascular entity.

Of note, ischemic lesions were predominant, whereas CMBs and WMH were generally mild burden with minimal progression in our DADA2 cohort. Previous studies reported a frequency of ischemic lesions ranging from 58.3% to75% in all DADA2 patients [18, 19, 21]. In our cohort with neurologic involvement, ischemic lesion was presented in all patients. Most patients showed multiple ischemic lesions compatible with recurrent clinical events. The ischemic lesions (symptomatic or clinically silent) demonstrated selective localization in strategic neurovascular territories, primarily affecting the brainstem (43.5%), thalamus (15.2%), internal capsule (14.5%), and basal ganglia (13.0%). Consistent with previous research [18, 19, 21], lesions were typically small, implicating a primary involvement of small deep perforating vessels [19]. Remarkably, while 32.6% of radiologically evident lesions corresponded to symptomatic stroke events, a substantial proportion (67.4%) remained asymptomatic, possibly reflecting subclinical microinfarcts in distal territories. The high prevalence of asymptomatic ischemic lesions suggests that cerebrovascular injury in DADA2 is likely underrecognized and may also occur in patients without reported neurological events. These findings support the implementation of routine neuroimaging evaluation in all DADA2 patients to enable early detection of silent cerebrovascular involvement and prompt initiation of anti-TNF therapy. Chronic inflammation, blood-brain barrier (BBB) disruption, and ischemia-hypoperfusion drive WMH progression [22–24], while inflammation and BBB damage correlate with CMB burden [25, 26]. The static nature of these two CSVD markers in our cohort challenges their centrality in DADA2-related vasculopathy. This dissociation implies a unique pathophysiology in which ischemic lesions arise primarily from transient TNF-α-mediated acute thromboinflammatory cascades rather than chronic endothelial or BBB dysfunction, which is further corroborated by lesion stabilization post anti-TNF therapy observed both in the present cohort and previous reports [9–11]. Deuitch et al. [27] demonstrated perivascular TNF deposition in the skin of DADA2 patients with a variety of clinical symptoms and severity, indicating a persistent, low-grade proinflammatory milieu even in the absence of overt systemic inflammation. Taken together, these findings support a dual-component inflammatory mechanism in DADA2 vasculopathy: a sustained background of low-degree inflammation and endothelial dysfunction that establishes vascular susceptibility, upon which episodic inflammatory exacerbations trigger acute endothelial injury and focal vascular occlusion. This framework reconciles the frequent occurrence of subclinical infarcts during clinically quiescent phases with the relative scarcity of WMH and CMB on neuroimaging. Specifically, chronic endothelial activation may predispose to subclinical microinfarcts, while more intense, transient inflammatory cascades account for clinically overt strokes.

Cerebral atrophy, particularly infratentorial volume loss, represents a cardinal neuroimaging feature of DADA2. While previous studies reported widely variable atrophy prevalence (3%−58.5%) in predominantly pediatric cohorts [15, 18, 19, 21], the present adult-focused cohort revealed a significantly higher rate of 69.2%, characterized by two distinct morphological patterns. Moreover, the results indicated that atrophy manifested as symmetric or asymmetric patterns, predominantly involving the brain stem, cerebellum, and cerebral peduncle. Accumulation of ischemic lesions and/or sub-radiological microinfarcts in the brain stem may underlie symmetrical infratentorial atrophy [20, 28]. Furthermore, we noticed that Wallerian degeneration could contribute to asymmetric atrophy of the brainstem, which may progress secondary to cerebrovascular events.

The above-mentioned DADA2 neuroimaging triad of predominant ischemic lesions, paradoxically low WMH and CMB burden, and selective infratentorial atrophy contrasts sharply with other CSVD [20, 29–31], reinforcing its distinct pathogenesis. First, WMH constitutes the earliest and most prevalent neuroimaging hallmark of cerebral autosomal dominant arteriopathy with subcortical infarcts and leukoencephalopathy (CADASIL), cerebral autosomal recessive arteriopathy with subcortical infarcts and leukoencephalopathy (CARASIL), and HTRA1-related autosomal dominant CSVD, which could even be detected in advanced-stage CADASIL cases devoid of lacunar infarcts [30–32]. Although pontine infarcts and rare CMBs are common to both pontine autosomal dominant microangiopathy and leukoencephalopathy (PADMAL) and DADA2, PADMAL exhibits significantly more severe WMH [33]. The minimal progression of WMH in DADA2 aligns with the proposed dual-component inflammatory model, in which transient TNF-α-driven endothelial inflammatory hits arise on a chronically vulnerable endothelium but do not typically induce sustained BBB dysfunction or chronic hypoperfusion, thus constraining the development of WMH. Second, unlike hypertensive CSVD (deep CMB clusters) or CAA (lobar CMB predominance) [20, 29], DADA2 exhibits rare CMBs without locational predilection, further suggesting microvascular fragility during inflammatory bursts rather than chronic lipohyalinosis or Aβ deposition related microvascular injury. Third, although both subcortical and cortical atrophy may result from CSVD [20, 34], our findings reveal that adult DADA2 patients exhibit a unique spatial atrophy pattern localized to infratentorial structures, implicating region-specific microvascular vulnerability to inflammatory injury. Altogether, this episodic mechanism diverges from relentless endothelial decay in arteriolosclerotic or other genetic CSVD, explaining both the scarcity of chronic WMH/CMBs and the predominance of acute ischemic lesions. Moreover, the dissociation between marked atrophy and minimal chronic CSVD markers further underscores acute inflammatory vasculopathy as the disease driver.

Therapeutic insights further validate this model. Pathophysiology evidence indicated that while perivascular TNF was present in DADA2, anti-TNF treatment reduced inflammation and rescued endothelial cell damage [27]. Anti-TNF therapy achieved 100% clinical/radiological stabilization by suppressing thromboinflammatory cascades [9–11], yet failed to reverse established atrophy—a dissociation highlighting TNF-α's role in acute vasculopathy but not chronic neurodegeneration. This dual effect mirrors the "two-hit" mechanism of the disease: acute hits (TNF-α-driven thrombosis) vs. cumulative hits (irreversible tissue loss from microinfarction).

Longitudinal assessments yielded two pivotal insights. First, partial acute infarcts exhibited volumetric regression or even complete resolution during follow-up. Potential explanations include underestimation of small lesions on conventional MRI, gliotic “healing” [19], or penumbral reversibility post-recanalization [35, 36]. Second, chronic ischemic lesions appearing hyperintense on T2WI and hypointense on T1WI with or without hyperintense on FLAIR on 3.0 T MRI, were confirmed as lacunes on 5.0 T MRI. This underscores the underestimation of lesion burden by conventional imaging and highlights the critical importance of advanced imaging modalities in precisely mapping DADA2-related cerebrovascular pathology.

Additionally, hemorrhagic lesions were uncommon in DADA2 with normal angiographic results. Hemorrhagic lesions were less frequent than ischemic lesions, with intracranial hemorrhagic stroke reported in about 4%−33% of DADA2 patients with varied clinical spectrum in previous research [10, 18, 19, 37]. In our cohort of DADA2 patients with neurological involvement, macroscopic symptomatic hemorrhagic lesions were observed in 46.2%, of which intracranial hemorrhagic lesions account for 38.5%. This higher prevalence likely reflects the selected nature of our cohort, which was enriched for patients with symptomatic neurological involvement. Reports regarding the location of hemorrhagic lesions remain controversial. Bulut et al. [18] reported hemorrhagic stroke primarily localized to the basal ganglia. However, a review from Dzhus et al. [21] showed that lobar hemorrhagic strokes occurred in approximately 7.1% of patients, more frequent than in deep, occurring in 2.4% of patients. In our patients, the prevalence of lobar macroscopic hemorrhage lesions was equal to hemorrhage in deep. Moreover, spinal hemorrhage was also observed. Patients with hemorrhagic lesions demonstrated increased susceptibility to fever and hematological involvements in our cohort. Nevertheless, the underlying causes of intracranial hemorrhagic stroke were still unclear in most reports, as in our patients [10, 18, 19, 37]. Despite the ischemic burden, conventional angiography frequently appears normal [1, 38], implying predominant small vessel pathology undetectable by current angiographic resolution. Due to the predominant feature of DADA2 involving small- and medium-sized arteries [16, 17], angiographic abnormalities were uncommon in our cases as in previous research [18]. Although multiple intracranial microaneurysms and localized stenosis of arteries were observed, the angiographic abnormalities were unresponsible for their clinical events. Geraldo et al. [19] reported eccentric vessel wall thickening and enhancement in all their cases, especially at the origin of brain stem perforators. However, this phenomenon was not observed in our research.

Our study still has some limitations that are inherent to research on ultra-rare disorders. Despite our efforts to ensure the representativeness of the sample, the sample size remains relatively small due to the rarity of the disease, which may potentially introduce some bias into the results. However, to the best of our knowledge, our present study is currently the largest cohort describing the neuroimaging features in DADA2 patients. Moreover, the present research was retrospective in design. Although participants were enrolled from a prospective cohort, the analysis itself remains subject to the constraints of retrospective review, including possible selection bias. Future prospective studies with larger sample sizes are needed to validate our findings. In addition, the atrophy was evaluated visually by experienced neurologists as in previous studies, which may bring subjective biases. However, all the neuroimaging data were evaluated by two experienced neurologists with good interobserver agreement, thereby ensuring the reliability of the results. Future work incorporating quantitative volumetric analyses will provide more objective data on brain volume change. Finally, it should be noted that direct pathological evidence was not available in this study. The proposed pathophysiology is therefore inferred indirectly from clinical and radiological observations and should be interpreted with caution. Future prospective research with larger, multi-center cohorts and correlative pathophysiological data will be essential to validate these mechanistic insights presented here.

## Conclusion

In summary, the present study demonstrated a profoundly elevated risk of stroke recurrence in DADA2, with approximately two-thirds of ischemic lesions being clinically silent. Critically, the initiation of anti-TNF therapy resulted in complete stroke cessation, underscoring its importance in disease management. The present study further delineates a distinctive neuroimaging phenotype in DADA2, characterized by three core features: (1) strategic ischemic vulnerability clustered in the brainstem and deep gray matter, (2) paradoxically low burdens of WMH and CMBs, and (3) prevalent infratentorial atrophy likely resulting from cumulative ischemic injury, subclinical/subradiological microvascular thromboinflammation, and Wallerian degeneration. The identification of this imaging triad provides a clinical decision-making scaffold, facilitating early diagnostic workup and targeted therapeutic intervention in suspected DADA2 cases. These findings may also suggest a pathophysiological divergence from other CSVDs, positioning inflammation-mediated microvascular injury as a defining hallmark of DADA2 vasculopathy. Further mechanistic studies targeting this neuroimaging-genetic signature might unveil novel therapeutic strategies to mitigate progressive neurovascular degeneration in DADA2.

## Supplementary Information

Below is the link to the electronic supplementary material.

Supplementary file1 (DOCX 1022 KB)

## Data Availability

Additional data related to this paper may be available upon reasonable requested from the corresponding author.
