# Supplementary material for: Decoding neurovascular signatures: advanced imaging insights in DADA2-Related Cerebral Microangiopathy
Source: Neurol Sci. 2025 Dec 19;47(1):18. doi: 10.1007/s10072-025-08676-9 (PMC12714772; doi:10.1007/s10072-025-08676-9)
Supplement: Supplementary file 1 — Supplementary file1 (DOCX 1022 KB) [file 10072_2025_8676_MOESM1_ESM.docx]

**Decoding Neurovascular Signatures: Advanced Imaging Insights in DADA2-Related Cerebral Microangiopathy**

**Journal name: Neurological Science**

**Authors: Yaping Zhou^1^,** **Min Shen^2^, Nan Jiang^1^, Hanhui Fu^1^, Fei Han ^1^, Yi-Cheng Zhu^1^, Ming Yao^1^* and** **Jun Ni^1^***

***Corresponding Author:**

**Dr. Ming Yao, MD PhD:** Department of Neurology, State Key Laboratory of Complex Severe and Rare Diseases, Peking Union Medical College Hospital, Chinese Academy of Medical Sciences and Peking Union Medical College, No 1, Shuaifuyuan, Dongcheng District, Beijing 100730, China. Email: pumchym2011@163.com

**Dr. Jun Ni, MD:** Department of Neurology, State Key Laboratory of Complex Severe and Rare Diseases, Peking Union Medical College Hospital, Chinese Academy of Medical Sciences and Peking Union Medical College, No 1, Shuaifuyuan, Dongcheng District, Beijing 100730, China. Email: pumchnijun@163.com

**Supplementary material**

**Table S1** Summary of *ADA2* genotypes and ADA2 enzyme activity of the enrolled patients.

**Table S2** Comparison of demographics and clinical features of DADA2 patients with and without hemorrhagic lesion.

**Fig. S1** Comparison of 3.0T and 5.0T MRI in identifying small lacune of DADA2

**Fig. S2** Temporal evolution of a resolved ischemic lesion

**Fig. S3** Temporal evolution of atrophy in DADA2.

Table S1. Summary of *ADA2* genotypes and ADA2 enzyme activity of the enrolled patients.

| **Patient No.** | **cDNA alteration** | **Amino acid alteration** | **Zygosity** | **ADA2 enzyme activity (U/L)** |
| --- | --- | --- | --- | --- |
| 1 | c.505C>G / Exon 6–8 del, 775 bp | p. Arg169Gly / - | Compound heterozygous | 0 |
| 2 | Exon 2–6 and Exon 8–10 del (heterozygous) / Exon 7 del (homozygous) | - | Compound heterozygous | 0 |
| 3 | c.1219G>T / c.334C>T | NR | Compound heterozygous | 0.1 |
| 4 | c.578C>T / c.1072G>A | p. Pro193Leu / p. Gly358Arg | Compound heterozygous | 0.1 |
| 5 | c.1065C>A / c.1147G>A | p. Phe355Leu / p. Gly383Ser | Compound heterozygous | 1.5 |
| 6 | c.1358A>G / c.505C>G | p. Tyr453Cys / p. Arg169Gly | Compound heterozygous | NR |
| 7 | c.139G＞C | p. Gly47Arg | Homozygous | 0.1 |
| 8 | c.1240G>A | p. Val414Met | Heterozygous | 0.1 |
| 9 | c.293del / c.505C>G | NR | Compound heterozygous | 0.1 |
| 10 | c.1072G>A / c.1065C>A | p. Gly358Arg / p. Phe355Leu | Compound heterozygous | 0.1 |
| 11 | c.1265_1267 del / c.139G>C | NR | Compound heterozygous | 0.2 |
| 12 | c.916C>T / Exon 7 del | p. Arg306Ter / - | Compound heterozygous | 0.4 |
| 13 | c.13G>C / Exon 7 del | p. Gly5Arg / - | Compound heterozygous | 0.2 |

Abbreviations: ADA2: adenosine deaminase 2, NR: not recorded.

| **Table S2** Comparison of demographics and clinical features of DADA2 patients with and without hemorrhagic lesion. | | |
| --- | --- | --- |
|  | With hemorrhagic lesions†  (n=6) | Without hemorrhagic lesion (n=7) |
| Age at onset (years, median, range) | 18 (0.5-26) | 14 (6-22) |
| Age at diagnose (years, median, range) | 23 (16-38) | 19 (16-27) |
| Time from onset to diagnose (years, median, range) | 12 (1-18) | 7 (0-16) |
| Age at neurological onset (years, median, range) | 16.5 (8-26) | 17 (7-22) |
| Males, n (%) | 4 (66.7) | 5 (71.4) |
| Fever, n (%) | 5 (83.3) | 4 (57.1) |
| Skin involvement |  |  |
| Livedo reticularis, n (%) | 5 (83.3) | 5 (71.4) |
| Skin/vulvar ulcers, n (%) | 1 (16.7) | 2 (28.6) |
| Raynaud's phenomenon, n (%) | 2 (33.3) | 1 (14.3) |
| Erythema modosum, n (%) | 2 (33.3) | 1 (14.3) |
| Hepatosplenomegaly, n (%) | 4 (66.7) | 6 (85.7) |
| Hypertension, n (%) | 2 (33.3) | 2 (28.6) |
| Hematological involvement*, n (%) | 3 (50.0) | 0 (0) |
| Laboratory results^#^, n (%) |  |  |
| Elevated ESR | 2 (33.3) | 3 (50.0) |
| Elevated CRP | 3 (50.0) | 5 (83.3) |

† Including 5 patients with intracranial hemorrhages and 1 patient with spinal hemorrhage.

* Including 1 patient with thrombocytopenia, leukopenia, and anemia, 2 patients with anemia and mild thrombocytosis.

^#^ Data were collected before anti-TNF therapy for 6 patients with and 6 without hemorrhagic lesions.

Abbreviations: DADA2: deficiency of adenosine deaminase 2, ESR: erythrocyte sedimentation rate, hsCRP: high sensitivity C-reactive protein, TNF: tumor necrosis factor.

**
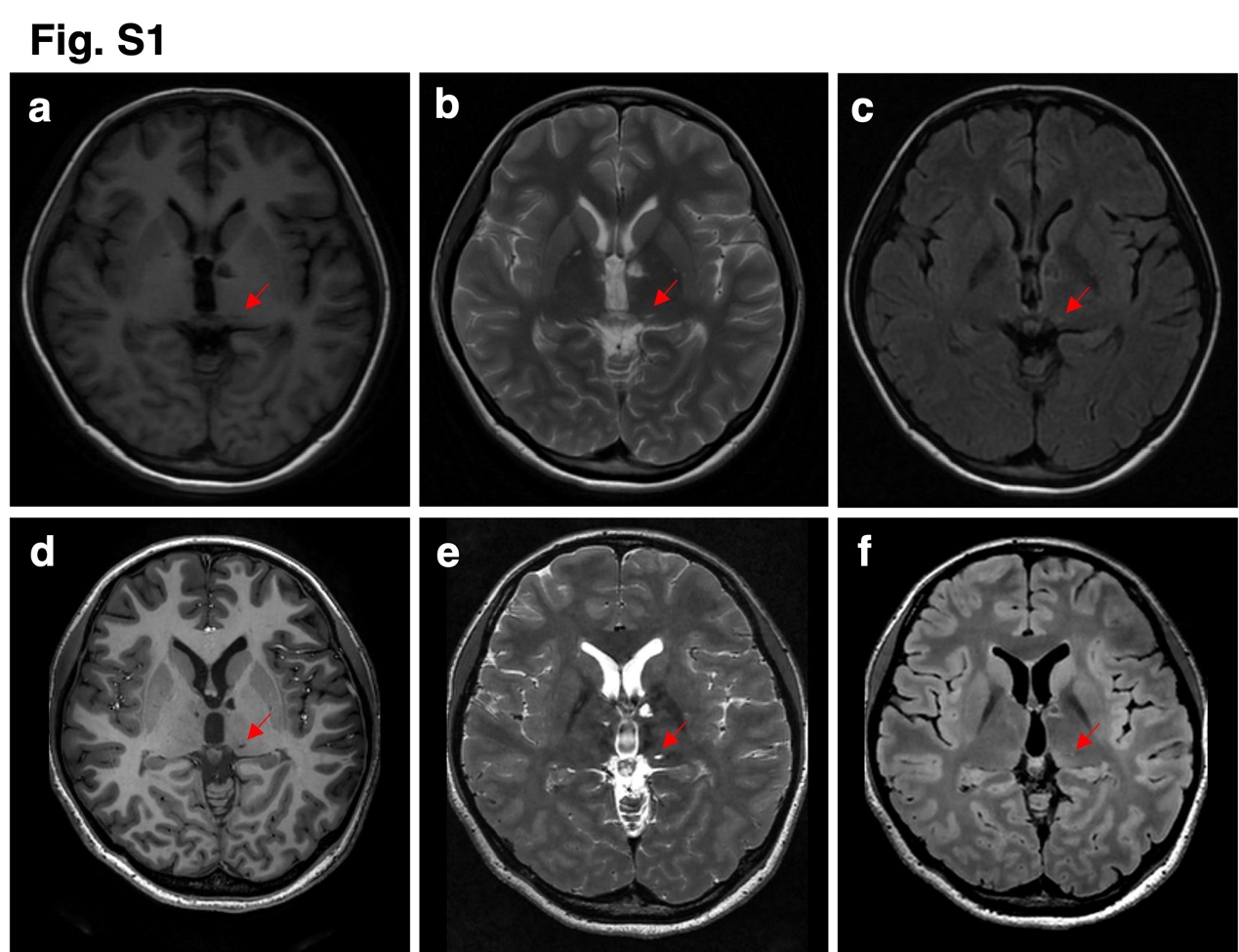
**

**Fig. S1** Comparison of 3.0T and 5.0T MRI in identifying small lacune of DADA2. a-c: (A 17-year-old female, before anti-TNF therapy) The small chronic ischemic lesion (arrow) was hypointense on T1WI (a), hyperintense on T2WI (b), and hyperintense on FLAIR (c) on 3.0T MRI, which was proven to be lacune on 5.0T MRI (d-f, the same patient, at the age of 19 years, 2 years after anti-TNF therapy). Specifically, the lesion was hypointense on T1WI (d), hyperintense on T2WI (e) and hypointense with a hyperintense rim on FLAIR (f).

**Abbreviations:** DADA2: Deficiency of adenosine deaminase 2, MRI: magnetic resonance imaging, T1WI: T1-weighted imaging, T2WI: T2-weighted imaging, FLAIR: fluid-attenuated inversion recovery imaging.

**
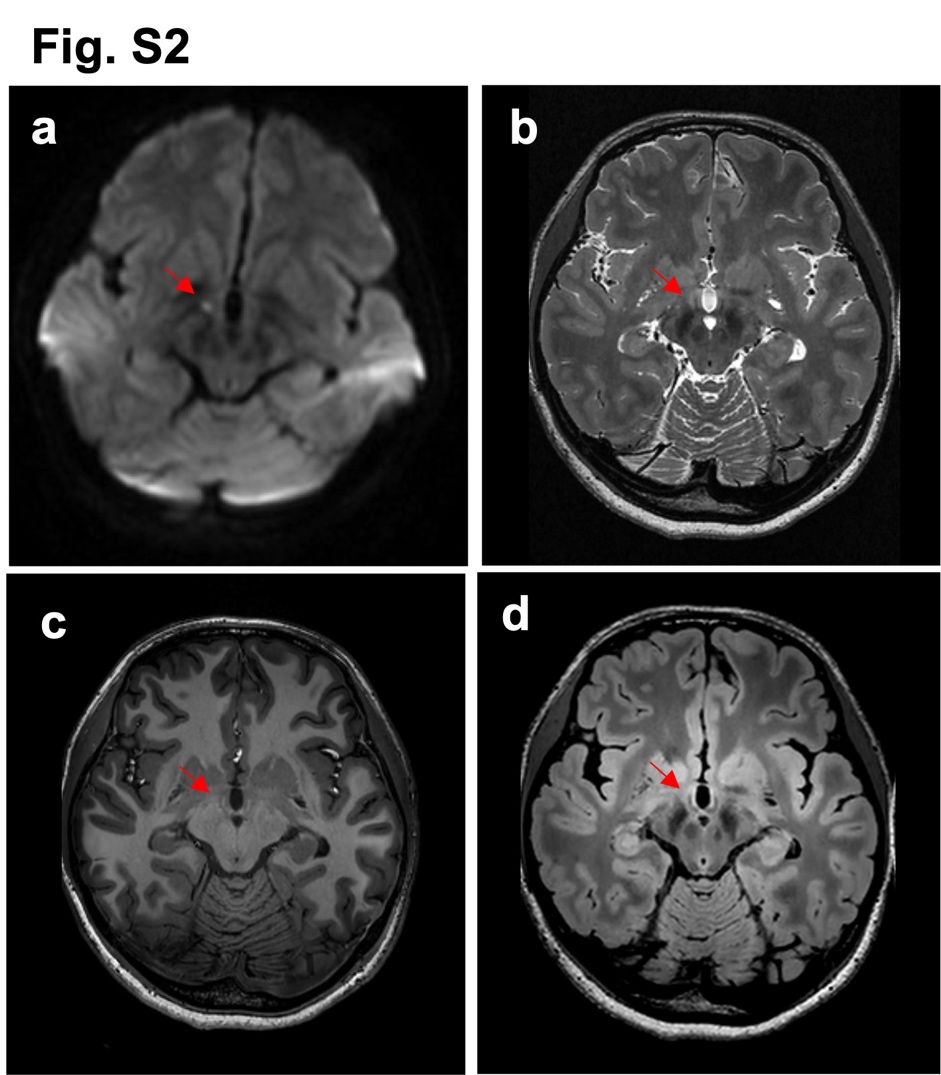
**

**Fig. S2** Temporal evolution of a resolved ischemic lesion. The DWI (a, 3.0T MRI) shows acute lacunar infarct (arrow), which was resolved 6 months after stroke (b: T2WI, c: T1WI, d: FLAIR, 5.0T MRI, a 17-year-old female, before anti-TNF therapy).

**Abbreviations:** DWI: diffusion-weighted imaging, FLAIR: fluid-attenuated inversion recovery imaging, MRI: magnetic resonance imaging, T1WI: T1-weighted imaging, T2WI: T2-weighted imaging.

**
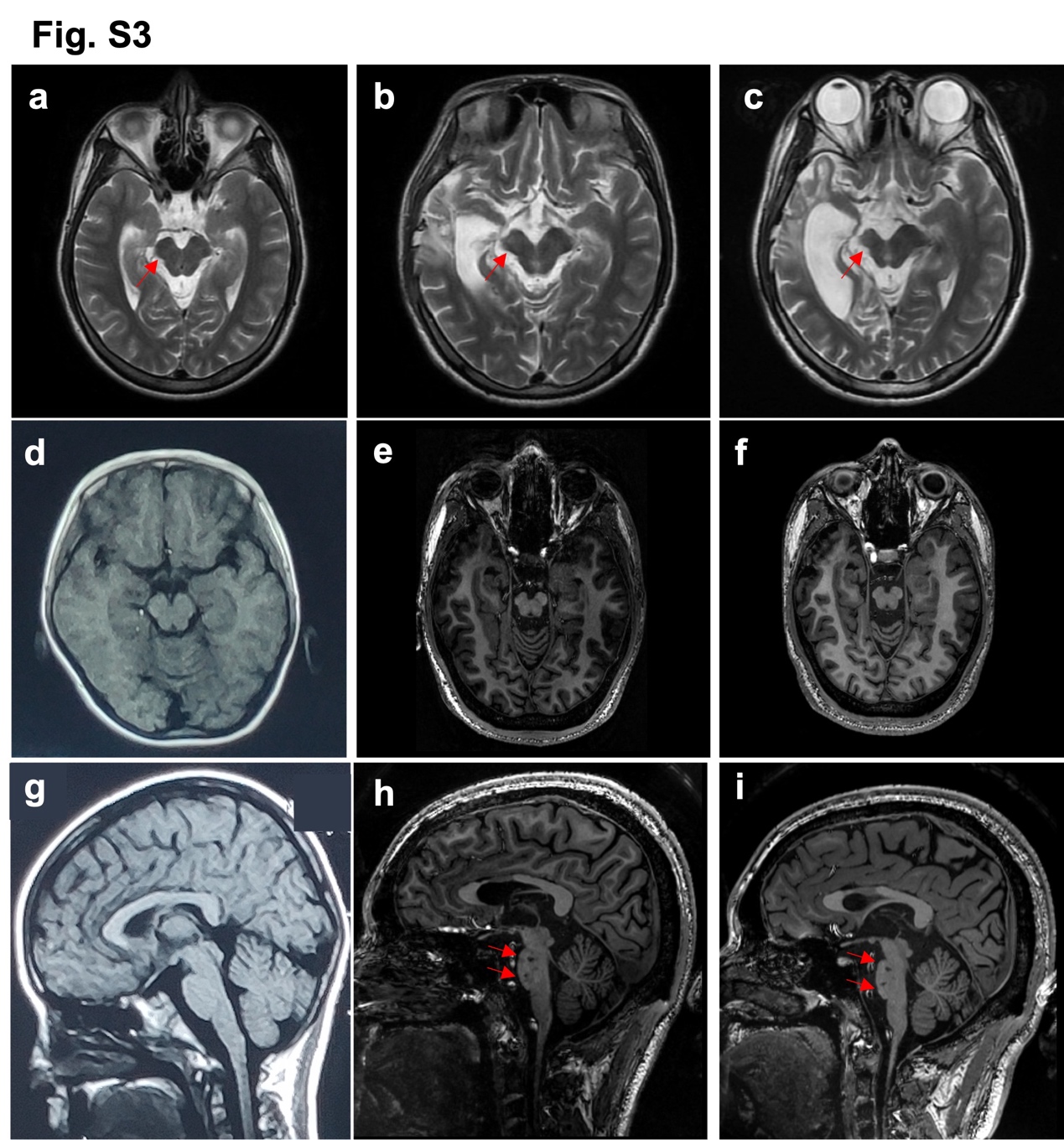
Fig. S3** Temporal evolution of atrophy in DADA2. a-c: The axial T2WI (3.0T MRI) before HS (a, 31-year-old male, before anti-TNF therapy), 4 months after HS (b, 31 years old, before anti-TNF therapy), and 1 year after HS(c, 10 months after anti-TNF therapy) show that the asymmetric atrophy of the right cerebral peduncle (arrow) was aggravated after HS. d-i: The axial and sagittal T1WI of a male patient at the age of 8 years (d, g, 3.0T MRI, before anti-TNF therapy), at the age of 23 (e, h, 5.0T MRI, 1 month after anti-TNF therapy), and at the age of 25 (f, i, 5.0T MRI, 2 years after anti-TNF therapy), respectively. The symmetric atrophy of the brainstem and cerebellum progressed with the accumulation of infarcts (arrow) in the brainstem (e, h) and was stable after anti-TNF therapy (f, i).

**Abbreviations:** DADA2: Deficiency of adenosine deaminase 2, FLAIR: fluid-attenuated inversion recovery imaging, HS: hemorrhagic stroke, TNF: tumor necrosis factor, MRI: magnetic resonance imaging, T1WI: T1-weighted imaging, T2WI: T2-weighted imaging.
